# Medical Convention of Ohio

**Published:** 1841-05

**Authors:** 


### MEDICAL CONVENTION OF OHIO.

A domestic affliction in the month of March, caused us to forget the preparation, for the last number of the Journal, of a notice of the meeting of this Convention on the 12th inst. at Columbus. It will be the fourth held by the physicians of Ohio, and will, we hope, be more numerously attended than either of the others. D.

